# Supra-organismal interactions in the human intestine

**DOI:** 10.3389/fcimb.2014.00047

**Published:** 2014-04-23

**Authors:** Laura Glendinning, Andrew Free

**Affiliations:** ^1^Developmental Biology, The Roslin Institute, University of EdinburghMidlothian, UK; ^2^School of Biological Sciences, Institute of Cell Biology, University of EdinburghEdinburgh, UK

**Keywords:** supraorganism, GI tract, microbiome, virome, dysbiosis

## Introduction

The term “supraorganism” (which we prefer to the more common but slightly less informative “superorganism”) refers to a collection of individuals which behave as a single unit with enhanced function. It was originally applied to groups of genetically-identical individuals such as social insect colonies (Moritz and Fuchs, 1998), but has since been expanded to include systems comprised of taxonomically-diverse species from all domains of life, as well as viruses (Salvucci, 2012). The human intestine plays host to up to 10^14^ bacteria, which outnumber the host's own cells by around an order of magnitude: microbial concentrations in the colon can reach 10^12^ cells per gram. There are also large numbers of viruses, predominantly bacteriophages, with at least 10^9^ viral particles present per gram of human feces. Current evidence presents a picture of prevailing homoeostasis between host, microbiome and virome consistent with the description of a supraorganism, which can nevertheless enter a disrupted alternative state termed “dysbiosis.” Here we review this evidence and the potential for the adoption of supra-organismal approaches toward the treatment and prevention of dysbiosis in the future.

## The intestinal microbiome—structure and function

The adult gastrointestinal (GI) tract harbors a vast and diverse population of microorganisms comprised of ~10^14^ individuals with a total genetic potential some two orders of magnitude larger than the host (Sommer and Bäckhed, 2013). Anaerobic Bacteria dominate this microbial community, which also includes aerobic or facultatively anaerobic Bacteria, Archaea, and Eukaryota, alongside a large but much more poorly-defined viral community (see below). Despite its diversity, the bacterial community comprises only a small subset of known phyla, suggesting strong selection of species adapted to the host environment and geared to a neutral or mutually-beneficial relationship with the host (Turnbaugh et al., 2009; The Human Microbiome Project Consortium, 2012). The phyla Firmicutes and Bacteroidetes dominate the gut microbiome, with members of the Proteobacteria, Actinobacteria, and Fusobacteria also abundant. The colonization of the infant GI tract begins at birth and initiates with facultatively anaerobic Proteobacteria, due to the initially oxidizing environment; anaerobic Bacteroidetes, Actinobacteria, and Firmicutes colonize later, and the microbiome composition stabilizes and begins to resemble that of the adult between 1 and 2 years of age (Sekirov et al., 2010).

**Figure 1:**
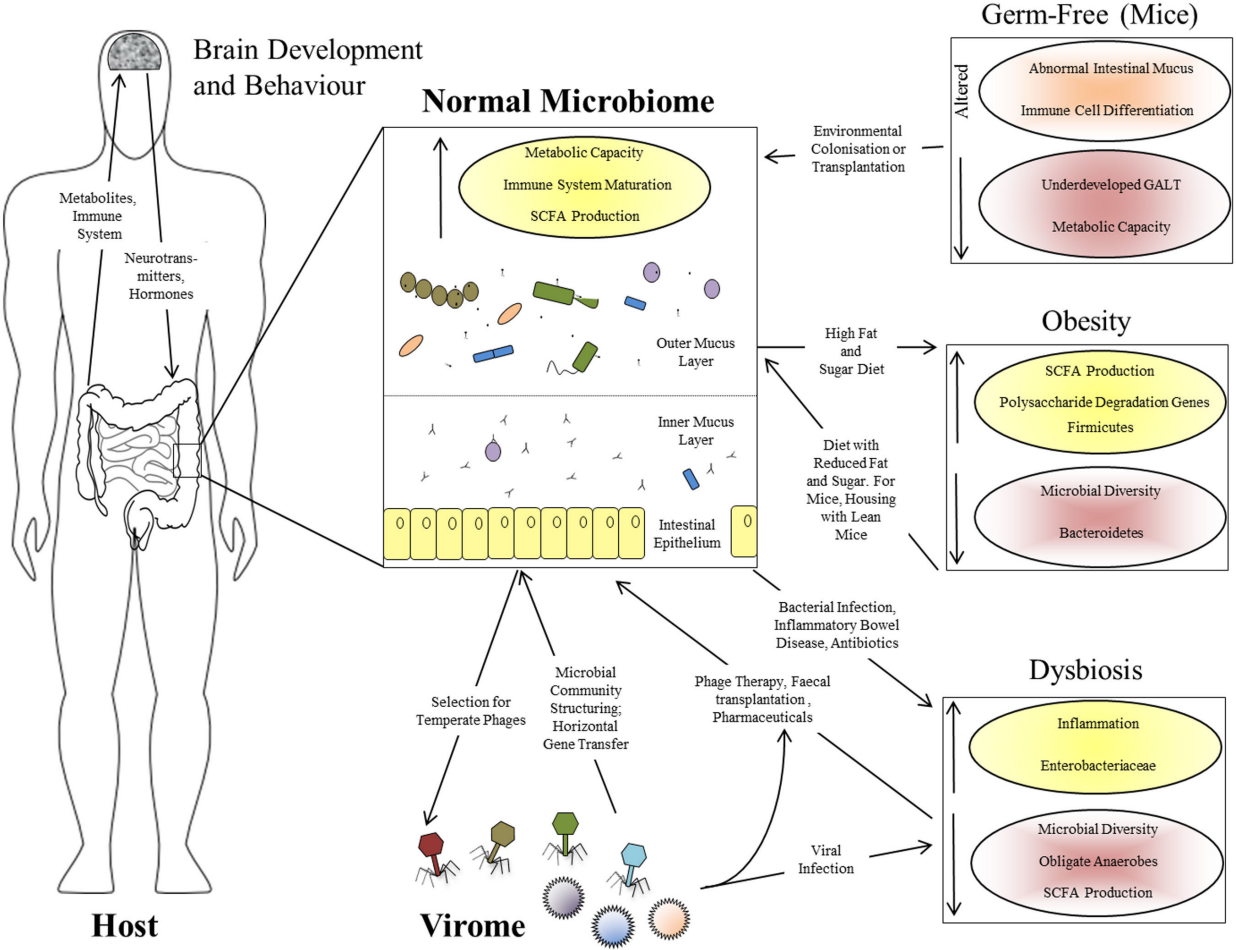
**Interactions between the human host, the intestinal microbiome, and the virome**. Functional effects of a normal intestinal microbiome are illustrated, together with the bi-directional interactions between the microbiome and virome, and between the GI tract and brain via the microbiome. The panels at the right show the perturbations associated with various altered microbiome states: the germ-free state in laboratory mice, obesity in mice and humans, and human dysbiosis. Increased parameters are shown with yellow shading and upward-pointing arrows; reduced parameters are shown with red shading and downward-pointing arrows.

That the gut microbiome is of immense benefit to its human host is attested to by its plethora of interactions throughout development (Figure 1), as well as the negative consequences of its disruption in dysbiosis (Walker and Lawley, 2013). Lederberg (2000) was the first to appreciate that the microbiome constitutes part of the human “self” in supraorganism theory. The best-defined contribution of the microbiota of the GI tract is a metabolic one: these microorganisms have a combined metabolic capacity equivalent to that of the liver, justifying their description as an additional human organ (Gill et al., 2006; Sommer and Bäckhed, 2013). Their key metabolic roles include the breakdown of otherwise indigestible polysaccharides into readily-absorbed short-chain fatty acids (SCFAs), the production of essential vitamins and other metabolites and the detoxification of harmful substances (Walker and Lawley, 2013). These activities all complement the host metabolism, expanding its functional capacity without the need for additional host-encoded genetic potential, while the other side of the mutualistic relationship is the constant temperature and nutrient-rich environment afforded to the microbiota by the host. The microbiota also plays a role in the metabolic phenotypes associated with diseases such as obesity: genetically obese (*ob*/*ob*) mice contain increased levels of SCFAs in their cecum, and their microbiota has an increased content of genes involved in polysaccharide degradation compared to that of lean mice (Tremaroli and Bäckhed, 2012), an observation replicated in humans (Turnbaugh et al., 2009). Even more strikingly, transplantation of the microbiota from an obese donor mouse to a germ-free recipient causes the recipient to gain fat at twice the level of a mouse transplanted with the microbiota from a lean donor (Turnbaugh et al., 2006), confirming that the microbiome can actively determine the phenotypic response of the host rather than merely responding passively to dietary intake. Furthermore, recipients transplanted with an “obese” microbiota fail to develop the obese phenotype if co-housed with mice harboring a “lean” microbiota. This phenomenon is associated with the transfer of specific Bacteroidetes species from the lean microbiota to the obese, but is dependent upon diet (Ridaura et al., 2013). There is therefore a complex interaction between host genome, microbiota, and environment in which all three components can play a controlling role (Figure 1).

The gut microbiome begins to assemble from birth and matures alongside the host immune system; it might therefore be expected to contribute significantly to the development of that system. The intestine is a unique, high-surface area, super-densely populated bioreactor with permeable walls which must retain biological integrity—the acquisition of an immune system which can tolerate the beneficial microbiota while responding effectively to pathogens must have been a crucial step in the evolution of the resulting supraorganism. This has led to the proposal that the adaptive immune system of vertebrates may have evolved to manage the complex communities of beneficial microorganisms found in their GI tracts, in contrast to the innate immunity and either very simple or well-contained microbiota of invertebrates (McFall-Ngai, 2007). Presumably this gave vertebrates the advantage of greater digestive flexibility, perhaps at the cost of susceptibility to autoimmunity. It is certainly true that the majority of the interactions of the immune system involve tolerance of the beneficial microbiota rather than antagonism toward pathogens (Arrietta and Finlay, 2012). Germ-free mice show underdevelopment of the gut-associated lymphoid tissue (GALT), altered differentiation of immune cell subtypes and an abnormal intestinal mucus layer which can be restored by treatment with bacterial lipopolysaccharide or peptidoglycan (Sommer and Bäckhed, 2013). The interaction between the immune system and the microbiota is therefore one of mutual reinforcement rather than top-down control (Figure 1).

Finally, the effects of the gut microbiome extend well beyond the GI tract itself. One emerging field is the interaction between intestinal microbiota and the brain via the so-called “gut-brain axis” (Collins et al., 2012). Such interactions include effects of gut microbiota on the levels of brain signaling molecules, dysbiosis-induced increase in anxiety-related behavior in mice, correspondingly reduced anxiety in germ-free mice (Sommer and Bäckhed, 2013) and a co-occurrence of psychiatric disorders with dysbioses such as irritable bowel syndrome (Hsiao et al., 2013). Therefore, bacterial by-products affect the brain directly or via endocrine cells or cytokines, while brain-derived hormones or neurotransmitters modulate the microbiota via gut physiology or influences on bacterial signaling and gene expression (Collins et al., 2012). These observations support a wider model in which the gut microbiota is an integrated component of the human body, with multiple, wide-ranging, and bi-directional interactions with many of its other components (Figure 1).

## A supra-organismal virome?

Viral abundance and diversity outnumbers that of bacteria in most ecosystems yet, with a few notable exceptions (Dinsdale et al., 2008), its characterization lags behind that of cellular microorganisms. The vast majority of viruses are bacteriophages which target prokaryotic cells, and much work on their effects on structuring the microbiota has been performed in marine systems. These studies suggest that bacteriophages have a major effect by targeting the fastest-growing bacterial species (the “Kill the Winner” hypothesis; Thingstad, 2000), causing a drastic restructuring of the microbial community (Bouvier and del Giorgio, 2007) and increasing its diversity (Hewson et al., 2003) by a process akin to negative frequency-dependent selection (Ayala and Campbell, 1974). However, studies of the human gut virome suggest that rather different bacterial-phage dynamics may exist therein. Although the virome shows greater interpersonal variation than the corresponding microbiome, intrapersonal diversity is low [a ~1:1 ratio of virotypes to bacterial phylotypes, compared to 10:1 in marine systems (Reyes et al., 2010)], virus particles and bacterial cells are present in similar numbers and most dominant virotypes are temporally stable and exhibit a temperate (non-lytic) lifestyle (Figure 1; Reyes et al., 2012). Such temporal variation as exists is largely restricted to lytic bacteriophages such as Microviridae, which evolve rapidly (>10^−5^ substitutions nucleotide^−1^ day^−1^), giving divergence of a level equivalent to the definition of novel viral species over a 2.5-year period (Minot et al., 2013). It is likely that a combination of bacterial strain-specific selection, long-term stability of the majority of the selected virome and rapid evolution of the remainder gives rise to the extreme interpersonal variability of the gut virome. More significantly, we propose that the contrast between the Lotka-Volterra dynamics of virus-bacteria interactions in marine systems and the apparent co-adaptation of the virome in the gut is indicative of a stronger supra-organismal organization in the latter.

The contribution of phage-encoded genes to bacterially-mediated ecosystem functions is well-documented in marine systems. Cyanophages infecting *Prochlorococcus* and *Synechococcus* species carry key functional genes, such as those encoding photosystem components, which can increase bacterial primary production (Rohwer and Thurber, 2009). Comparable effects of the gut virome on host function via virally-encoded genetic or metabolic potential are less clear-cut. Many of the best-defined examples are virulence factors, such as the phage 933W Shiga-like toxin (Plunkett et al., 1999), which assist invasion of the normal microbiota by bacterial pathogens; viruses are also implicated in the global spread of antibiotic-resistance genes via horizontal gene transfer (Smillie et al., 2011). However, there are indications that the virome may carry beneficial intestinal functions to host bacteria, such as the observation of many genes for anaerobic metabolism in fecally-derived viral contigs (Reyes et al., 2010) and the potential for modification of bacterial carbohydrate utilization via phage receptors (Reyes et al., 2012).

The gut virome of healthy individuals is massively dominated by bacteriophages, with eukaryotic virus sequences either undetectable or at very low levels (Minot et al., 2013). However, significant occurrences of eukaryotic enteroviruses are particularly associated with the host disease and microbiome disruption of dysbiosis, as discussed below.

## Host-microbiome-virome interactions and dysbiosis

“Dysbiosis” is a rather poorly-defined term which is generally taken to mean a severe compositional disruption of the (intestinal) microbiota usually associated with an effect on host health (Walker and Lawley, 2013). It is characterized by a decline in microbial diversity and in the prevalence of obligate anaerobes belonging to the Firmicutes phylum, often associated with a decline in Bacteroidetes and an increase in facultatively-anaerobic Proteobacteria. Known causes include infection with pathogens of the GI tract, broad-spectrum antibiotic treatment and host-initiated disruptions such as colorectal cancer (Walker and Lawley, 2013), but distinguishing between a causal and responsive role for the microbiome in conditions such as Crohn's disease and ulcerative colitis (UC) is often difficult.

Both analysis and, potentially, treatment of dysbiosis can benefit from a supra-organismal approach. Apart from the obvious host disruption caused by diarrhea, microbiome-dependent metabolic functions such as SCFA production are adversely affected in dysbiosis, and this can in turn exacerbate diarrhea and contribute toward chronic inflammation (Ramakrishna and Mathan, 1993). Inflammation and disruption of the epithelial mucosal barrier probably themselves prolong dysbiosis by favoring the attachment and survival of *Enterobacteriaceae* over anti-inflammatory Firmicutes species (Willing et al., 2011). The eukaryotic virome can also be involved in the onset and/or maintenance of dysbiosis: diarrhea caused by agents such as adenovirus, norovirus, and rotavirus is associated with microbiomes of reduced diversity, reduced Bacteroidetes levels and increased Proteobacteria (Ma et al., 2011; Nelson et al., 2012). There is therefore a complex set of causative and downstream interactions leading to the persistence of the dysbiotic state in the intestinal supra-organism (Figure 1).

There has been much recent interest in the use of microbiota transplantation, typically using healthy donor feces, to treat intestinal dysbiosis (Palmer, 2011). As well as clinical demonstrations of this approach in patients with recurrent *Clostridium difficile* infection and UC (Walker and Lawley, 2013), experiments in mouse models demonstrate that it is an effective way of re-establishing a healthy microbiome in dysbiotic animals which is resistant to subsequent challenge with pathogens (Lawley et al., 2012). The exciting prospect of using the bacteriophage components of the virome to structure a healthy microbiome and promote beneficial functions such as nutrient biosynthesis (Reyes et al., 2012) would be a logical extension of such an approach to dysbiosis treatment. Our rapidly-expanding knowledge of the supra-organism defined by the host intestine, its microbiome and virome should enable us to turn the possibility of such therapies into reality in the coming years. Moreover, the general approach of linking our knowledge of this supra-organism to ecological concepts relating to homoeostasis and the modulation of a habitat by its resident organisms (Jones et al., 1994; Odling-Smee et al., 2003; Dyke and Weaver, 2013) will be beneficial for our understanding of the ecosystem state changes in the GI tract that we can now monitor so readily.

### Conflict of interest statement

The authors declare that the research was conducted in the absence of any commercial or financial relationships that could be construed as a potential conflict of interest.
